# Podiatric Skin and Nail Involvement and Biomechanical Pathology in Renal Transplant Recipients: Assessment of the Foot as a Contributing Factor to Their Health

**DOI:** 10.3390/healthcare11233078

**Published:** 2023-11-30

**Authors:** Cristina González-Martín, Vanesa Balboa-Barreiro, Mª Teresa Garcia-Rodriguez, Raquel Veiga-Seijo, Teresa Seoane-Pillado, Estefanía Couceiro-Sanchez

**Affiliations:** 1 Research Group in Nursing and Health Care, Instituto de Investigación Biomédica de A Coruña (INIBIC), Complexo Hospitalario Universitario de A Coruña (CHUAC), Universidade da Coruña, Sergas, 15006 A Coruña, Spain; cristina.gmartin@udc.es (C.G.-M.); vanesa.balboa.barreiro@sergas.es (V.B.-B.); raquel.veiga.seijo@udc.es (R.V.-S.); maria.teresa.seoane.pillado@udc.es (T.S.-P.); 2 Department of Health Sciences, Faculty of Nursing and Podiatry, Universidade da Coruña, Campus Esteiro, 15471 Ferrol, Spain; estefaniacouceiro@gmail.com; 3 Research Group in Rheumatology and Health, Universidade da Coruña (UDC), 15006 A Coruña, Spain

**Keywords:** foot, renal transplant, podiatry, health, skin pathology, nail pathology

## Abstract

Although several studies show the prevalence of podiatric conditions in people with end-stage renal disease or renal replacement therapy with hemodialysis, there is little scientific literature on this when subjects are undergoing kidney transplantation. The aim of this study is to determine the prevalence of podiatric skin and nail pathology in renal transplant recipients. A descriptive, observational, prevalence study was conducted at the Nephrology Department of the University Hospital of A Coruña. A total of 371 subjects were studied. The variables studied were sociodemographic (age, sex), anthropometric (Body Mass Index), comorbidity (Charlson Comorbidity Index), and podological (skin and nail alterations). A high presence of skin (83.1%) and nail pathology (85.4%) was observed, with hyperkeratosis (68.8%), onychogryphosis (39.4%), and onychocryptosis (36.9%) being the most predominant alterations. Although it was not significant, patients with a higher risk of presenting podiatric pathology were of female sex and had a high BMI, and both age and the Charlson comorbidity index were significantly associated with this risk. There was an increased risk of both skin and nail pathology at older age and in the presence of diabetes mellitus.

## 1. Introduction

Chronic kidney disease (CKD) is defined as a decrease in kidney function or the presence of persistent kidney damage for at least three months, constituting a major public health problem today [1,2,3]. In recent decades, the incidence of its most severe manifestations, known as end-stage renal disease (ESRD), has increased, as has the need for other treatments such as renal transplantation [3]. The study carried out by Gorostide et al. [4] on the prevalence of chronic kidney disease in Spain observed that this disease affected 15.1% of the population, being more frequent in men (23.1% vs. 7.3% in women), its prevalence increased with age (4.8% in subjects aged 18–44 years, 17.4% in subjects aged 45–64 years, and 37.3% in subjects ≥65 years), and was more frequent in patients with cardiovascular pathology (39.8% vs. 14.6% in subjects without cardiovascular pathology). In addition, 1% would need replacement treatment (dialysis or kidney transplantation) due to the progression of the disease to advanced stages.

As for the prevalence of podiatric pathology in the group of kidney transplant patients, this has not been studied so far. However, ESRD is increasingly common in older people, especially those with diabetes or hypertension. In this sense, it is known that the prevalence of podiatric pathology in the population over 65 years ranges between 71% and 90.7% [5,6]. As for suffering from a systemic disease, problems in the feet such as skin lesions, plantar helomas, neuropathy, and ulcers have been identified as harmful complications associated with ESRD and diabetes mellitus (DM) [7,8]. Subjects on hemodialysis, and especially those with diabetes, are susceptible to hypertension and peripheral vascular disease, placing them at risk when developing foot problems and therefore resulting in a high incidence of morbidity and mortality [9,10]. Likewise, peripheral neuropathy in subjects with ESRD, as in DM, is frequently present, caused, among others, by uremia and vasculitis [10].

Although several studies show the prevalence of podiatric conditions in people with ESRD or renal replacement therapy with hemodialysis, there is little scientific literature on this when subjects are treated with kidney transplantation [9,10,11]. In addition, factors such as the progressive aging of the population, the increase in the prevalence of diabetes mellitus, hypertension, or obesity, as well as the increase in the prevalence of CKD pose an added risk when developing foot conditions [6,7,8,9,12]. Therefore, the objective of this study was to determine the prevalence of skin and nail podiatric pathology and the variables associated with its presence in a sample of kidney transplant patients.

## 2. Materials and Methods

A descriptive study was carried out within a prospective cohort of people who have received a kidney transplant at the University Hospital of A Coruña (HUAC) in the period 1981–2020.

The established criteria for the subjects included in this study were: recipients of a kidney transplant with a functioning graft; those who attended consecutive outpatient consultations in Nephrology, and those who gave their informed consent to participate in this study voluntarily. People with lower limb amputations or impaired ambulation were excluded from the research.

During the data collection period (from January 2021 to September 2022), a total of 371 subjects were studied through consecutive sampling. Podiatric information was available in *n* = 272 subjects. This sample size allows for estimating the parameters of interest with a certainty of 95% and an accuracy of ±6%. (for podiatric variables, *n* < 0.05).

The variables studied were: sociodemographic (year of birth, sex), anthropometric (Body Mass Index (BMI)), medical variables (date of kidney transplant, diabetes mellitus, perimalleolar edema, Godget’s sign), comorbidity (Charlson Comorbidity Index), podiatric pathology variables (plantar footprint, cutaneous, nail, and biomechanical pathology), and quality of life (SF-36 questionnaire, End-Stage Renal Desease Symtom Check-list Transplantation Module, Foot Function Index, Foot Health Status Questionnaire).

The patient’s associated comorbidity was studied using the Charlson Comorbidity Score [13]. It encompasses 19 medical situations weighted from 1 to 6, with results ranging from 0 to 37. This index, described in 1987, makes it possible to predict mortality at one year. Regarding the interpretation of the index, it is generally considered absence of comorbidity: 0–1 points, low comorbidity: 2 points, and high comorbidity: >3 points. At prolonged follow-ups (>5 years), the prediction of mortality should be corrected for the age factor. This correction is made by adding one point to the index for each decade after the age of 50.

The study and knowledge of the morphology of the plantar footprint are of great interest for the classification and diagnosis of some foot pathologies. The pedigraph was used for its analysis. Once the pedigraphs have been obtained, the footprints will be categorized as flat or normal.

The cutaneous and nail pathology at the foot level will be assessed through an observational clinical examination developed by a specialized podiatrist. In the case of skin conditions, they will be inspected both on the dorsal and plantar aspects of the foot, including interdigital spaces. Pathologies such as keratopathy, hyperhidrosis, fungal and wart affections, and pruritus will be included. Among the nail conditions, onychocryptosis, onychogryphosis, onychomycosis, Muehrcke’s lines, half and half nails, and Terry’s nails, among others, will be assessed. Perimaleolar edema will also be assessed, establishing whether it presents itself or not by Godget’s sign (when pressing on an area of the foot, the skin remains sunken) as positive or negative. Finally, peripheral neuropathy has been studied, which has been quantified through the Neuropathy Disability Score (NDS) [14,15], which has included the clinical evaluation of pain, vibration, sensitivity to temperature (cold/heat), and osteotendinous reflexes.

Finally, to determine quality of life, different questionnaires were passed, such as the SF-36. This questionnaire was developed in the early 1990s by Ware et al. as a generic instrument for measuring Health-Related Quality of Life (HRQoL), applicable to both patients and healthy populations [16]. It contains 36 items covering 8 dimensions (physical function, physical role, body pain, general health, vitality, social function, emotional role, and mental health). The scores of the 8 dimensions of the SF-36 are ordered in such a way that the higher the value, the better the state of health. Another questionnaire that was included in this study was the End-Stage Renal Disease Symptom Checklist-Transplantation Module (ESRD-SCL) [17], which is one of the few validated instruments that exist for the measurement of HRQoL in kidney transplant recipients. It consists of 43 items grouped into 6 dimensions (physical capacity limitations, cognitive capacity limitations, cardiac and renal dysfunction, corticosteroid side effects, excessive hair and hair growth, and psychological distress associated with transplantation). All items are scored on a 5-point Likert scale, ranging from 0 (not at all) to 4 (very much), and a high score is indicative of a worse HRQoL.

As a foot-specific quality of life questionnaire, the Foot Function Index (FFI) is a clinical tool created and validated in 1991 by Budiman-Mak, Conrad, and Roach [18]. It consists of 23 items divided into 3 sub-scales (pain, disability, and activity limitation). To be completed, the questionnaire presents a visual analogue scale divided into 10 equal segments ranging from 0 (minimum score) to 10 (maximum score). Higher scores indicate worsening foot health and poor foot-related quality of life. Finally, the Foot Health Status Questionnaire (FHSQ) was passed [19,20], which assesses pain, functional capacity, footwear, and overall foot health. The questionnaire contains 13 items that assess 4 domains of foot health (pain, function, footwear, and general foot health). Each question has several answers, and these form a Likert ordinal scale. Scores range from 0 (worst foot health) to 100 (best foot health), so higher scores reflect better foot health and quality of life.

The current ethical-legal aspects of the development of this research have been respected, as evidenced by the favorable report of the Clinical Research Ethics Committee of Galicia (2013/155). Each study participant provided signed informed consent once the study information sheet was read, and the appropriate doubts were resolved by the researchers.

Regarding the statistical analysis, a descriptive analysis of the variables included in this study was performed. The qualitative variables were expressed as an absolute value and as a percentage after the assessment of the 95% confidence interval, formulating it in a frequency table. Quantitative variables were expressed as mean ± standard deviation. The comparison of means was carried out by means of Student’s *t* or Mann–Whitney test as appropriate after checking normality with the Kolgomorov–Smirnov test. The association of qualitative variables was estimated by means of the Chi-square statistic. Finally, logistic regression models were used to determine the association of different variables with each other.

## 3. Results

The present study included 371 people with kidney transplantation, with a mean age of 48.07 ± 12.49 years at the time of transplantation (Table 1). The sample was dominated by males (65.5%), 42.3% overweight, and 22.1% obese. Among the pathologies studied, the most frequent in this sample were diabetes (24.3%), of which 65.5% were post-transplant, and perimalleolar edema (20.5%). Obtaining an average score of the age-adjusted Charlson Index of 2.16 ± 1.73.

The most prevalent skin pathology was hyperkeratosis (68.8%), followed by the symptom of pruritus (13.2%). Among nail pathologies, the most predominant were onychogryphosis (36.5%) and onychocryptosis (31.4%). Regarding the biomechanical pathology, we observed that Hallux Abductus Valgus (HAV) was the most prevalent alteration, reaching 58.9% of cases and being bilateral in 78.9%. This prevalence was followed by flat feet (45%), being bilateral in 62.3% of cases, claw toes (41.8%) being found in both feet in 88.5% of cases, and Hallux Extensus (34.2%) with 78.7% in both feet. (Table 2). Most patients (72.4%) had both pathologies (cutaneous and nail), and 94.9% had at least one of the two pathologies included in this study.

In the sample studied, it was observed that patients with cutaneous and nail pathology (Table 3) were older than those who did not present any of the pathologies (49.76 ± 12.62 vs. 42.79 ± 12.20 years; *p* < 0.001). There was a tendency toward a higher risk of presenting podiatric pathology among women (OR = 1.26) and a higher BMI; these differences were not significant. Regarding comorbidity, a higher Charlson index was observed (1.07 ± 1.19 vs. 0.75 ± 1.14; OR = 1.29) among patients with both skin and nail alterations, and a higher prevalence of diabetes (28.6% vs. 13.2%; *p* = 0.007). Other pathologies not included in the Charlson index were also more prevalent among patients with both podiatric alterations, such as perimalleolar edema, Godget, or peripheral neuropathy, the latter with a 3-fold higher risk in patients with cutaneous and nail alteration compared to patients with a single alteration or none (OR = 3.03). Adjusting for age and sex, it was observed that having diabetes significantly increased the risk of suffering from both podiatric pathologies in at least one of the lower limbs (OR = 2.17, 95% CI = 1.02–4.60).

Regarding the factors associated with the presence of cutaneous and nail pathology (Table 3), it was observed that both age at the time of transplantation (OR = 1.07) and comorbidity index (OR = 1.29) were significantly associated with this risk. The presence of diabetes, perimalleolar edema, Godget, or peripheral neuropathy also had a significant impact. Adjusting for age at the time of transplantation, sex, and comorbidity, it was observed that the only variables with a significant impact on the risk of presenting skin and nail pathology were age and diabetes. Thus, the older the age, the greater the risk of suffering both pathologies (OR = 1.04, 95% CI = (1.02–1.07)), and having diabetes mellitus multiplied this risk by 2.17.

A multivariate regression analysis was performed, adjusting for sex, age at transplant, and diabetes, and it was observed that both age and diabetes are variables related to the appearance of skin and nail pathologies. When segmenting the models by patients with and without diabetes, it is observed that in patients with diabetes, being female and age increase the probability of this pathology, although not significantly, while in patients without diabetes, only age has a significant effect (Table 4 and Table 5).

Since the most frequent pathology was HAV, patients with this pathology were older at the time of transplantation than those patients in whom this podiatric alteration was not observed (49.30 ± 12.04 vs. 46.31 ± 12.93 years), had greater comorbidity according to the Charlson index (2.33 ± 1.71 vs. 1.93 ± 1.73), and had a lower BMI (Table 6). A higher risk of presenting the alteration studied was observed in females, but it was not significant (OR = 1.33, 95% CI = 0.86–2.07). Regarding podiatric characteristics, it was observed that having flat feet (OR = 1.88) and claw toes (OR = 2.57) was associated with the presence of Hallux Valgus (HV) (OR = 1.75 and 1.51, respectively).

A multivariate logistic regression analysis was performed to identify the joint effect of sociodemographic and anthropometric variables on the presence or absence of HV. A model with the covariates age, sex, Charlson index, flat feet, and claw toes was adjusted to study the impact of these factors on the probability of presenting the alteration of interest. All of them were identified as risk factors. The risk of presenting the pathology under study increases with age (OR = 1.04). The risk of HAV is 2.64 times higher in females than in males. Having flat feet increases the likelihood of HV by five times, but not significantly. Claw toes are an independent risk factor for HV (OR = 2.11, 95% CI: 1.30; 3.43).

The results of foot pathology on the individual’s functionality were studied using the Foot Function Index (FFI) questionnaire (Table 7). The overall score of the questionnaire was low (5.61 ± 13.95 points), and 50% of the patients surveyed obtained a score of 0. In the descriptive score according to the sub-scales (pain, disability, and functional limitation), there is a lot of variability, but in all three cases, a median value equal to 0 points is obtained. In the Foot Health Status Questionnaire (FHSQ), an instrument for measuring quality of life related to specific foot health, the results obtained showed a good quality of life in the domains of pain, functionality, and footwear, with the highest score in the domain of foot function (94.73 ± 16.28). The domain with the lowest scores was general foot health, with a mean score of 45.25 ± 22.77 points. In the questionnaire used to assess health-related quality of life in kidney transplant recipients, it was observed that the dimension that achieved the highest score (worst quality of life) was limitations of physical capacity (0.89 ± 0.71), followed by limitations of cognitive capacity (0.75 ± 0.52). Lower scores were observed, which is related to better quality of life, in corticosteroid side effects and excessive hair and hair growth (Table 7).

## 4. Discussion

This work constitutes an investigation that tries to cover the knowledge gaps identified in the literature in relation to the skin and nail repercussions in the lower limb from a podiatric perspective in people who have been recipients of a kidney transplant. 

The scientific evidence is ample in relation to the repercussions on the foot in subjects with ESRD, Diabetes Mellitus, and the elderly; however, there has not been extensive and current literature that identifies the repercussions on the foot when the subjects have been recipients of a kidney transplant. Despite this, there are articles that highlight the importance of identifying skin and nail conditions, as it helps reduce the association of the person’s health status with morbidity and mortality [21], since in many cases these manifestations are generally secondary to immunosuppressive treatment [22].

### 4.1. General Characteristics of the Sample Studied

Regarding the results of the present study, the mean age was 56.01 ± 11.39, being higher than that found in other studies, which did not reach an average of 50 years [23,24]. With regard to sex, the majority of the sample studied were men (65.5%), which has similar sampling characteristics to the studies by Prakash et al. [23], Dicle et al. [24], or Garrido & Borges-Costa [25]. While the mean age at transplantation was 48.07 ± 12.49 years, the mean BMI was 27.00 ± 4.93 kg/m^2^, the prevalence of diabetes was 24.3%, of which 15.9% occurred after transplantation, and the prevalence of peripheral neuropathy was 16.4%. In the study by Martínez-Mier et al. [26], the mean age and mean BMI found in the sample were 30 ± 10.07 years (range 18–68) and 24 ± 4.05 kg/m^2^ (range 14–36.9), respectively. This difference in mean BMI may be due to the background of the study population (ethnicity, diet, age, etc.), with the age range of our study being the widest (8.56–74.09). The passage of time between the time of the transplant and the patient’s examination may be another variable to be taken into account, as several studies have shown the existence of an increase in weight after transplantation, which increases over time [27,28].

The prevalence of peripheral neuropathy found in our sample was 16.4%, somewhat lower than that reported by the scientific literature consulted. Mohammadi MH et al. [29], in a recent systematic review and meta-analysis on neurological complications after kidney transplantation, found that the most common neurological disorder in these patients was peripheral neuropathy, reaching a prevalence of 29%. On the other hand, Sobhani S et al. [30] and Shiferaw et al. [31] reported a prevalence of 53% (range from 16% to 87%) and 46% (range from 7.5% to 83.4%) in their respective meta-analyses. The lack of unified criteria for diagnosing peripheral neuropathy, the age of the participants in each study, the duration and severity of diabetes, as well as the response rate of the study population and early treatments in developed countries, account for the variation in the data reported in the existing scientific literature.

### 4.2. Prevalence of Biomechanical Pathology

The highest prevalence of biomechanical pathology was Hallux Abductus Valgus (HAV) (58.9%), flat feet (45%), and claw toes (41.8%). As there are no specific studies of biomechanical foot pathologies in kidney transplant patients, the comparison of the data found in our study was compared with the data reported in the literature in healthy populations or populations affected by other pathologies. Thus, the prevalence of HAV in the study by Menz et al. [32] ranged from 21% to 65%, the range within which our study is located. Regarding the prevalence of flat feet, most of the studies consulted show lower prevalences than in our study [33,34]. In this regard, it is important to note that several researchers have reported inconsistent values for the prevalence of flat feet among the adult population. This can be attributed to the different methods used to assess the flexibility of the arches of the foot, in addition to the lack of accurate clinical or radiographic criteria for defining flat feet [33,35]. Finally, the prevalence of claw toes was similar to other studies. Rojas-Villarraga et al. [36] studied foot alterations in a sample of 95 patients with rheumatoid arthritis, finding a prevalence of claw toes of 39%. Likewise, Vázquez-Navarrete et al. [37] reported a prevalence of claw toes of 41% in a sample of 100 adults over 60 years of age, associating comorbidities such as osteoarticular disease with the presence of claw toes.

### 4.3. Prevalence of Skin and Nail Pathology

In relation to the data identified in the articles on cutaneous and nail lesions, difficulties have been found in locating studies that address these health problems specifically in the lower limb, thus making it difficult to contrast our results with those of other research. In the first place, in relation to cutaneous conditions, a high prevalence of them was observed (83.1%), constituting a high percentage of hyperkeratosis (68.8%) of the total sample studied. Despite this incidence, no similar studies have been found to compare and contrast these results.

Nail pathology has also been a relevant health problem in the patients studied, since 85.4% have presented it. The Prakash et al. [23] study found a much lower prevalence than in our study, of 7.4%. This may be because this study had a much smaller sample size (*n* = 54). However, a recent study carried out by Ankudowicz et al. [21] in 2018 found a prevalence more similar to that of our research (56.6% of nail pathology), although not as high since the patients were not kidney transplants but were undergoing hemodialysis treatment. 

Specifically, both onychogryphosis (39.4%) and onychocryptosis (36.9%) stood out among the nail pathologies observed in our work. The study by Dicle et al. [24] identified that 5.7% of the sample (*n* = 220) had onychomycosis, a percentage that differs from that of our research (22.0%). In this sense, Lima et al. [22] identified that the most prevalent fungal condition was onychomycosis (24.5%), a percentage more like that of our study, which was 22.0%. Other studies [38,39] concluded that the prevalence rate of onychomycosis and leukonychia was higher in kidney transplant patients than in the healthy population, with statistically significant differences. 

Another nail and skin pathology that appears in transplant patients and that may be influenced by immunosuppressive treatment is mycosis. In this study, the presence of onychomycosis was 22% in at least one foot, and although the patients have not been followed up or immunosuppressive treatment has been taken into account, authors such as Pereiro et al. [40], in their article on the most frequent mycoses in immunosuppressed patients, determine that the most common mycoses in patients with solid organ transplantation are cryptococcosis, aspergillosis, and candidiasis. Among the risk factors would be the first 6 months after transplant due to the fact that the dose of immunosuppressants at this time is higher.

In relation to the sociodemographic characteristics, the disease of the participants studied, and the fact of presenting or not presenting cutaneous and nail pathology, it is noteworthy that there were significant differences in terms of age at examination and transplantation, BMI, the fact of suffering from Diabetes Mellitus before and after transplantation, perimaleolar edema, Godget’s sign, and peripheral neuropathy. Thus, the high prevalence of skin and nail diseases is responsible for the morbidity added to the health status of kidney transplant recipients [25]. In addition, the results of research conducted by Moloney et al. [41] show how suffering from these conditions has a significant impact on the quality of life of this group. All this reveals the importance of regularly evaluating the cutaneous and nail status of the foot [42], thus promoting prevention and reducing the possibility of deterioration in the health status of this group. 

### 4.4. Functionality, Quality of Life, and Pathology of the Foot

With the results obtained, it can be stated that the presence of skin and nail pathology of exogenous origin will negatively modify both the quality of life considering the foot measured with the FHSQ and the functionality of the foot measured with the FFI, except for nail pathology in the latter case. as its presence does not modify the functionality of the foot measured with the FFI.

Authors such as Garrow et al. [43] not only found a relationship between the pathology described and quality of life considering the foot, but also described how the presence of skin pathology in the foot modifies the quality of life measured with the SF-36. On the other hand, Pérez-García [44] found that both skin and nail pathology of exogenous origin decreased the quality of life, taking into account the foot (FHQS) and the functionality of the foot (FFI).

Despite the fact that this study has been carried out as rigorously as possible, the authors acknowledge the existence of weaknesses, among which are the failure to follow up with the patients after the performance of the kidney transplant, the failure to take into account immunosuppressive medication, or the non-inclusion of a control group. Taking this into account would answer questions such as determining the influence that transplantation has on podiatric pathology or the connection that skin and nail pathologies have on graft function. Despite this, the results obtained are considered important since it is the first time that the prevalence of podiatric pathology in these patients has been determined. Based on these preliminary results, future lines of research will be established that include variables that can provide answers to the questions previously exposed.

## 5. Conclusions

This study shows the high incidence of cutaneous and nail pathology in lower limbs suffered by kidney transplant patients, with older patients, greater comorbidity, and a higher BMI presenting more podiatric alterations. In summary, it is concluded that:

1. In the sample of kidney transplant patients studied, more than 90% of patients had at least one of the podiatric pathologies under study, and seven out of ten had both.
2. The most prevalent cutaneous and nail pathologies were hyperkeratosis, pruritus, onychogryphosis, and onychocryptosis, while the biomechanical pathologies were Hallux Abductus Valgus, flat feet, and claw toes.
3. Patients with cutaneous and nail pathology were older, had a higher BMI, and had comorbidities such as diabetes, peripheral neuropathy, and perimalleolar edema.
4. The risk of suffering from both cutaneous and nail pathologies increases with age, as does the presence of diabetes after adjusting for sex.
5. The presence of skin and nail pathology of exogenous origin will negatively modify the quality of life.

## Figures and Tables

**Table 1:** Distribution of patients according to different variables studied.

Variables				
	*n*	Media ± SD	Median	Range
Age at exploration (years)	371	56.01 ± 11.39	57	24–81
Age at transplant (years)	371	48.07 ± 12.49	49.45	8.56–74.09
BMI (kg/m^2^)	371	27.00 ± 4.93	26.49	16.22–56.01
Age adjusted CCI	371	2.16 ± 1.73	2	0–9
Unadjusted CCI	371	0.91 ± 1.17	0	0–6
	*n*	%	CI 95%	
Sex				
Male	243/371	65.5%	(60.53; 70.47)	
Female	128/371	34.5%	(29.53; 39.47)	
Categorized BMI				
Underweight (BMI <18.5 kg/m^2^)	2/371	0.5%	(0.07; 1.93)	
Normal Weight (18.5 kg/m^2^ ≤ BMI < 25 kg/m^2^)	130/371	35%	(30.05; 40.03)	
Overweight (25 kg/m^2^ ≤ BMI < 30 kg/m^2^)	157/371	42.3%	(37.16; 47.48)	
Obesity (BMI ≥ 30 kg/m^2^)	82/371	22.1%	(17.75; 26.46)	
Diabetes Mellitus				
No	281/371	75.7%	(71. 25; 80.24)	
Yes	90/371	24.3%	(19.76; 28.76)	
Post-transplant	59/90	65.5%	(55.18; 75.93)	
Edema perimaleolar				
No	295/371	79.5%	(75.27; 83.75)	
Yes	76/371	20.5%	(16.24; 24.73)	
Godget				
Negative	311/371	83.8%	(79.95; 87.71)	
Positive	60/371	16.2%	(12.30; 20.05)	
Peripheral neuropathy				
No	310/371	83.6%	(79.65; 87.46)	
Yes	61/371	16.4%	(12.54; 20.35)	

SD: Standard Deviation; CI: Confidence Interval; BMI: Body Mass Index; CCI: Charlson Comorbidity Index.

**Table 2:** Description of cutaneous, nail, and biomechanical pathologies of the foot.

Variables					
Skin Pathology of Exogenous Origin					
		Left Foot		Right Foot	
		*n* (%)	CI 95%	*n* (%)	CI 95%
Type of alteration					
Hyperkeratosis		171 (62.9)	(56.9–68.8)	180 (66.2)	(60.4–72.0)
Heloma		28 (10.3)	(6.5–14.1)	33 (12.1)	(8.1–16.2)
Swallow		32 (11.8)	(7.8–15.8)	34 (12.5)	(8.4–16.6)
Miliary Helomas		11 (4.0)	(1.2–6.6)	13 (4.8)	(2.1–7.5)
Simple maceration of the skin		26 (9.6)	(5.9–13.2)	34 (12.5)	(8.4–16.6)
Hyperhidrosis		4 (1.5)	(0.4–3.7)	4 (1.5)	(0.4–3.7)
Tinea pedis		6 (2.2)	(0.3–4.1)	6 (2.2)	(0.3–4.1)
Warts		7 (2.6)	(0.5–4.6)	6 (2.2)	(0.3–4.1)
Ulcers		3 (1.1)	(0.2–3.2)	1 (0.4)	(0.0–2.0)
Pruritus		36 (13.2)	(9.0–17.5)	36 (13.2)	(9.0–17.5)
**Nail pathology of exogenous origin**					
		**Left Foot**		**Right Foot**	
		***n* (%)**	**CI 95%**	***n* (%)**	**CI 95%**
Type of alteration					
Onychogrifosis		100 (36.5)	(30.6–42.4)	102 (37.2)	(31.3–43.1)
Onychocryptosis		86 (31.4)	(25.7–37.1)	87 (31.9)	(26.1–37.5)
Onycholysis		42 (15.3)	(10.9–19.8)	42 (15.3)	(10.9–19.8)
Onychomycosis		47 (17.2)	(12.6–21.9)	43 (15.8)	(11.3–20.3)
Beau’s Lines		69 (25.2)	(19.9–30.5)	60 (21.9)	(16.8–27.0)
Longitudinal grooves		61 (22.3)	(17.2–27.4)	62 (22.6)	(17.5–27.8)
Subungual hematoma		7 (2.6)	(0.5–4.6)	7 (2.6)	(0.5–4.6)
Splinter hemorrhage		45 (16.5)	(11.9–21.1)	34 (12.5)	(8.4–16.6)
Leukonychia		27 (9.9)	(6.2–13.6)	29 (10.6)	(6.8–14.5)
Muerhrcke Lines		26 (9.5)	(5.9–13.2)	26 (9.5)	(5.9–13.2)
Half and Half Nails		8 (2.9)	(0.8–5.1)	8 (2.9)	(0.8–5.1)
Terry’s Nails		30 (11.0)	(7.1–14.9)	30 (11.0)	(7.1–14.9)
**Biomechanical pathology**					
		**Left Foot**		**Right Foot**	
		***n* (%)**	**CI 95%**	***n* (%)**	**CI 95%**
Type of alteration					
Footprint	Normal	192 (51.8)	(46.5–57.0)	181 (48.8)	(43.6–54.0)
	Flat	113 (30.5)	(25.6–35.3)	158 (42.6)	(37.4–47.8)
	Sparkling wine	66 (17.8)	(13.8–21.8)	32 (8.6)	(5.6–11.6)
		**Unilateral**		**Bilateral**	
		***n* (%)**	**CI 95%**	***n* (%)**	**CI 95%**
Flatfoot		63 (37.7)	(30.1–45.4)	104 (62.3)	(54.6–69.9)
Hallux Abductus Valgus		46(21.1)	(15.5–26.8)	172 (78.9)	(73.3–84.6)
Hallux Rigidus		14 (45.2)	(26.0–64.3)	17 (54.8)	(35.7–74.0)
Hallux Extensus		27 (21.3)	(13.8–28.8)	100 (78.7)	(71.2–86.3)
Claw Fingers		18 (11.5)	(6.2–16.9)	138 (88.5)	(83.1–93.8)

CI: Confidence Interval.

**Table 3:** Characteristics of the patients included, depending on the presence or absence of skin and nail pathology.

	Cutaneos and Nail Pathology				
	No	Yes			
	Media ± SD	Media ± SD	*p*	OR (CI 95%)	Adjusted OR (CI 95%)
Age at exploration (years)	49.38 ± 12.14	57.42 ± 10.57	<0.001	1.07 (1.04; 1.09)	
Age at transplant (years)	42.79 ± 12.20	49.76 ± 12.62	<0.001	1.04 (1.02; 1.06)	1.04 (1.02; 1.06)
Unadjusted CCI	0.75 ± 1.14	1.07 ± 1.19	0.018	1.29 (1.00; 1.67)	
Age-adjusted CCI	1.51 ± 1.64	2.42 ± 1.71	<0.001	1.40 (1.17; 1.67)	
BMI (kg/m^2^)	26.20 ± 5.31	27.35 ± 4.93	0.012	1.05 (0.99; 1.12)	
	*n* (%)	*n* (%)	*p*		
Sex			0.881		
Male	50 (65.8)	129 (64.8)		1	1
Female	26 (34.2)	70 (35.2)		1.26 (0.70; 2.28)	1.23 (0.69; 2.22)
Categorized BMI			0.152		
Normal Weight or Infraweight (<25 kg/m^2^)	33 (43.4)	62 (31.2)		1	
Overweight (25 kg/m^2^ ≤ IMC < 30 kg/m^2^)	31 (40.8)	95 (47.7)		1.63 (0.91; 2.93)	
Obesity (IMC ≥ 30 kg/m^2^)	12 (15.8)	42 (21.1)		1.86 (0.86; 4.02)	
Diabetes Mellitus			0.007		
No	66 (86.8)	142 (71.4)		1	1
Yes	10 (13.2)	57 (28.6)		2.01 (0.94; 4.30)	2.17 (1.02; 4.60)
Post-transplant diabetes mellitus			0.036		
No	70 (92.1)	163 (81.9)		1	
Yes	6 (7.9)	36 (18.1)		2.58 (1.04; 6.39)	
Edema perimaleolar			0.038		
No	66 (86.8)	150 (75.4)		1	
Yes	10 (13.2)	49 (24.6)		2.16 (1.03; 4.51)	
Godget			0.048		
No	69 (90.8)	161 (80.9)		1	
Yes	7 (9.2)	38 (19.1)		2.33 (0.99; 5.46)	
Peripheral neuropathy			0.021		
No	71 (93.4)	164 (82.4)		1	
Yes	5 (6.6)	35 (17.6)		3.03 (1.14; 8.05)	

SD: Standard Deviation; CI: Confidence Interval; CCI: Charlson Comorbidity Index; BMI: Body Mass Index.

**Table 4:** Multivariate regression model adjusted for sex, age, and diabetes.

	B	SE	*p*	OR	95% IC (OR)
Age at transplant	0.040	0.011	0.000	1.041	1.018–1.064
Sex (woman)	0.210	0.299	0.481	1.234	0.687–2.216
Diabetes	0.773	0.384	0.044	2.166	1.020–4.600
constant	−1.118	0.547	0.041	0.327	

**Table 5:** Regression models were segmented by patients with and without diabetes.

	Patients with Diabetes				
	B	SE	*p*	OR	95% IC (OR)
Age at transplant	0.038	0.028	0.174	1.039	0.983–1.097
Sex (woman)	0.734	0.851	0.388	2.084	0.393–11.040
constant	−0.371	1.429	0.795	0.690	
	**Patients without diabetes**				
Age at transplant	0.040	0.012	0.001	1.041	1.016–1.066
Sex (woman)	0.128	0.323	0.692	1.136	0.0604–2.139
constant	−1.093	0.597	0.067	0.335	

**Table 6:** Characteristics of the patients were included according to the presence or absence of Hallux Abductus valgus.

	Hallux Abductus Valgus						
	No	Yes					
	Media ± SD	Media ± SD	*p*	OR	CI 95%	Adjusted OR	CI 95%
Age at exploration (years)	53.4 ± 11.6	57.9 ± 10.9	0.000	1.04	1.02; 1.06	1.04	1.01; 1.06
Age at transplant (years)	46.3 ± 12.9	49.3 ± 12.0	0.023	1.02	1.00; 1.04		
Unadjusted CCI	0.9 ± 1.2	0.9 ± 1.2	0.855	1.02	0.85; 1.21	0.93	0.76; 1.14
Age-adjusted CCI	1.9 ± 1.7	2.3 ± 1.7	0.020	1.14	1.01; 1.29		
BMI (kg/m^2^)	27.8 ± 5.9	26.5 ± 4.0	0.073	0.95	0.91; 0.99	0.89	0.85; 0.95

CI: Confidence Interval; SD: Standard Deviation CCI: Charlson Comorbidity Index; BMI: Body Mass Index.

**Table 7:** Quality of life and functionality of the foot.

Quality of Life Questionnaires	*n*	Media ± SD	Median	Range
Foot Function Index (FFI)	371	5.6 ± 14.0	0	0–86
Pain	371	9.4 ± 20.7	0	0–95
Disability	371	4.7 ± 15.7	0	0–80
Limitation	371	1.8 ± 8.5	0	0–80
Foot Health Status Questionnaire (FHSQ)				
Foot Pain Domain	371	89.4 ± 15.3	93.8	18.8–100
Foot Function Domain	371	94.7 ± 16.3	100	0–100
Footwear Domain	371	72.1 ± 35.6	91.7	0–100
General Health Foot Domain	371	45.3 ± 22.8	50.0	0–100
SF-36				
Summary Physical Index	370	44.7 ± 9.2	46.7	15.7–67.4
Summary Mental Index	370	49.6 ± 11.3	52.8	6.3–69.7
End-stage Renal Disease Symptom check-list Transplantation Module (ESRDS-TM)				
Physical capacity limitations	371	0.9 ± 0.7	0.7	0.0–6.1
Cognitive ability limitations	371	0.8 ± 0.5	0.6	0.0–2.8
Renal and cardiac dysfunction	371	0.6 ± 0.5	0.6	0.0–0.6
Corticosteroid side effects	371	0.4 ± 0.5	0.2	0.0–2.4
Overgrowth of vello and hair	371	0.4 ± 0.5	0.2	0.0–3.2
Psychological distress associated with transplantation	371	0.6 ± 0.6	0.4	0.0–3.3

SD: Standard Deviation.

## Data Availability

Data are contained within the article.
